# Impending Airway Threat in a Neonate: Intralesional Sclerotherapy As Salvage Therapy for Giant Congenital Cervical Cystic Hygroma

**DOI:** 10.7759/cureus.101809

**Published:** 2026-01-18

**Authors:** Muhammad Mudasir Saleem, Habib Rehman, Hira Shamim, Ismail Mazhar, Anaab Wasim, Mir Rai, Fareha Azam, Momina Ahmed, Faheem Ullah

**Affiliations:** 1 Department of Paediatric and General Surgery, Combined Military Hospital Lahore, Lahore, PAK; 2 Department of Surgery, Combined Military Hospital Multan, Multan, PAK; 3 Department of Paediatric Surgery, Combined Military Hospital Peshawar, Peshawar, PAK; 4 Department of Internal Medicine, CMH Lahore Medical College and Institute of Dentistry, Lahore, PAK; 5 Department of Paediatric Surgery, Lahore Medical and Dental College, Lahore, PAK; 6 Department of Internal Medicine, Lahore General Hospital, Lahore, PAK; 7 Department of Paediatric Surgery, CMH Lahore Medical College and Institute of Dentistry, Lahore, PAK

**Keywords:** airway compromise, bleomycin sclerotherapy, cervical cystic hygroma, giant cervical mass, intralesional sclerotherapy, lymphatic malformation, minimally invasive therapy, neonatal airway obstruction, neonatal emergency management, ultrasound-guided intervention

## Abstract

Cervical cystic hygroma, or lymphatic malformation, is a rare congenital anomaly that can present as a life-threatening airway emergency in neonates. Prompt recognition and timely management are critical for survival. While surgical excision has traditionally been the mainstay of treatment, extensive lesions involving vital neck structures carry significant operative risks in the neonatal period. We report a full-term neonate presenting at birth with a giant cervical cystic hygroma causing airway compression and respiratory distress. Due to high surgical risk, emergency ultrasound-guided intralesional bleomycin sclerotherapy was performed, resulting in a marked reduction in lesion size. At the six-month follow-up, there was no recurrence. This case underscores the role of image-guided intralesional sclerotherapy as a safe, effective, and minimally invasive life-saving alternative for neonates with giant cervical cystic hygroma.

## Introduction

Cystic hygroma is a benign congenital anomaly of the lymphatic system, arising from failure of the lymphatic sacs to connect with the venous system [1]. It occurs in approximately 1 in 12,000 live births and most commonly involves the posterior triangle of the neck [2]. Large cervical cystic hygromas can exert pressure on adjacent structures, leading to airway distortion, feeding difficulties, and, in neonates, potentially life-threatening respiratory compromise [3]. Prompt airway management and timely intervention are therefore essential for survival. Although surgical excision has traditionally been considered the definitive treatment, it carries significant risks, most notably incomplete resection, as well as hemorrhage and nerve injury, particularly in large or infiltrative lesions [4]. Intralesional sclerotherapy with agents such as bleomycin or OK-432 has emerged as a safe, effective, and minimally invasive first-line alternative, particularly for extensive or inoperable lesions [5].

Cystic hygromas can be macrocystic, microcystic, or a combination of both, and this classification affects treatment decisions. In this case, the macrocystic type responded well to sclerotherapy, which is often preferred in neonates with large or high-risk lesions because it lowers the chances of airway problems and surgical complications. However, there is still limited information on managing extensive macrocystic cervical cystic hygromas in high-risk newborns, underscoring the challenge highlighted by this case.

## Case presentation

A newborn male was referred from a peripheral health facility immediately after an emergency cesarean section, following inadequate fetal surveillance during pregnancy, with a large neck swelling noted at birth. The infant arrived at our emergency department without oxygen support and exhibited no signs of respiratory distress. A nasogastric tube had been placed for feeding. No detailed records were available regarding antenatal care or immediate postnatal management. The baby tolerated feeds via the nasogastric tube and had passed meconium. On admission, he was pink on room air with stable vital signs.

**Figure 1:**
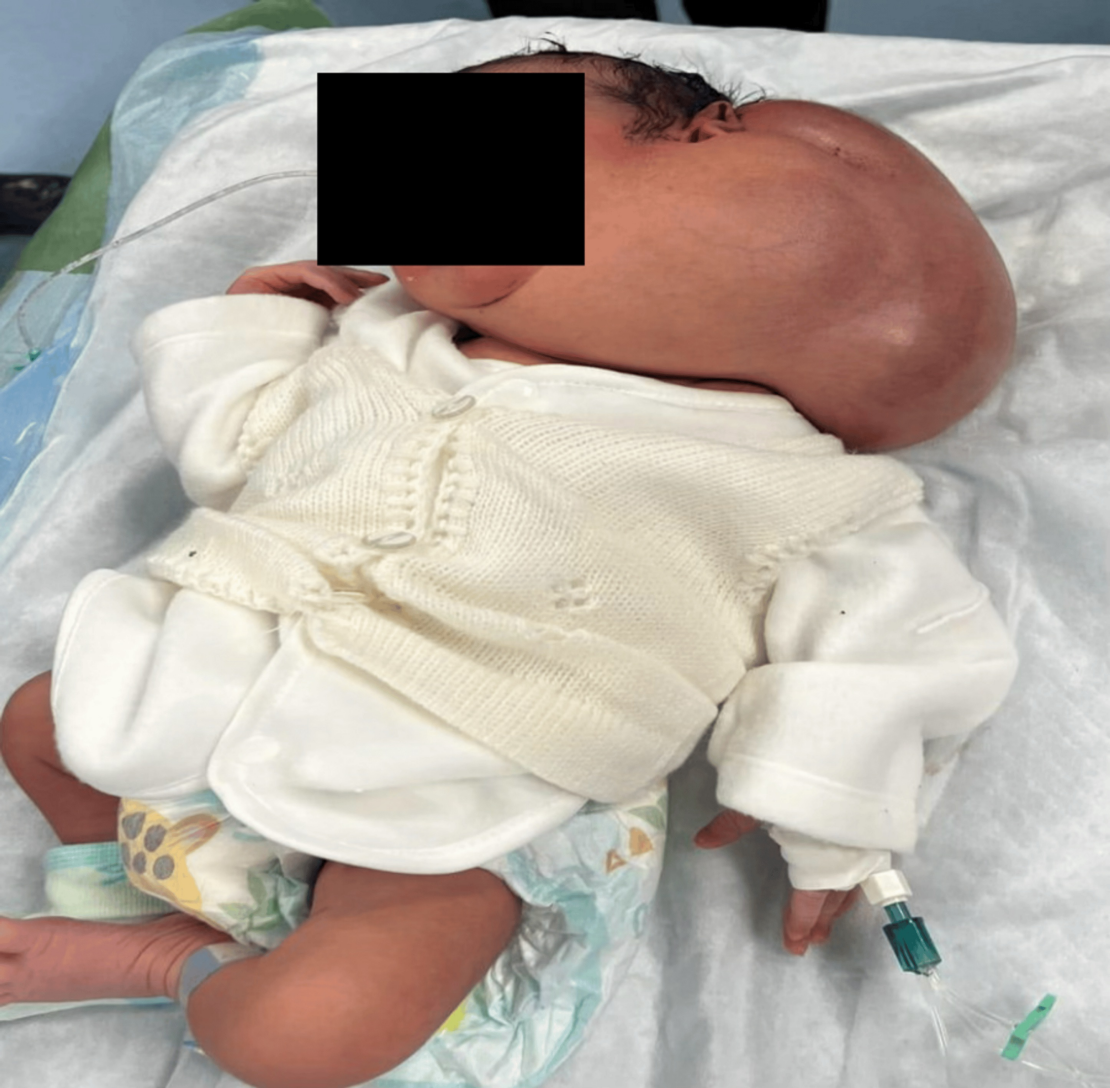
Left-sided neck swelling on initial presentation.

Examination of the neck revealed a massive, soft, cystic swelling measuring approximately 30 × 40 cm on the left side, extending from the angle of the mandible to the clavicle and crossing the midline posteriorly. The overlying skin was stretched and shiny, without dilated veins or ulceration. The swelling caused marked neck asymmetry and lateral displacement of the head toward the contralateral side (Figure 1).

A diagnosis of a large congenital macrocystic cervical cystic hygroma was made based on clinical findings, with a high risk of airway compromise due to mass effect on adjacent structures. The parents were counseled in detail regarding the potential need for emergency intervention, and the infant was admitted to the neonatal intensive care unit for close monitoring, supportive care, and further evaluation. A CT scan of the neck was performed, revealing a multiloculated cystic lesion extending from the left posterior cervical region to the supraclavicular area, causing mass effect on adjacent structures.

**Figure 2:**
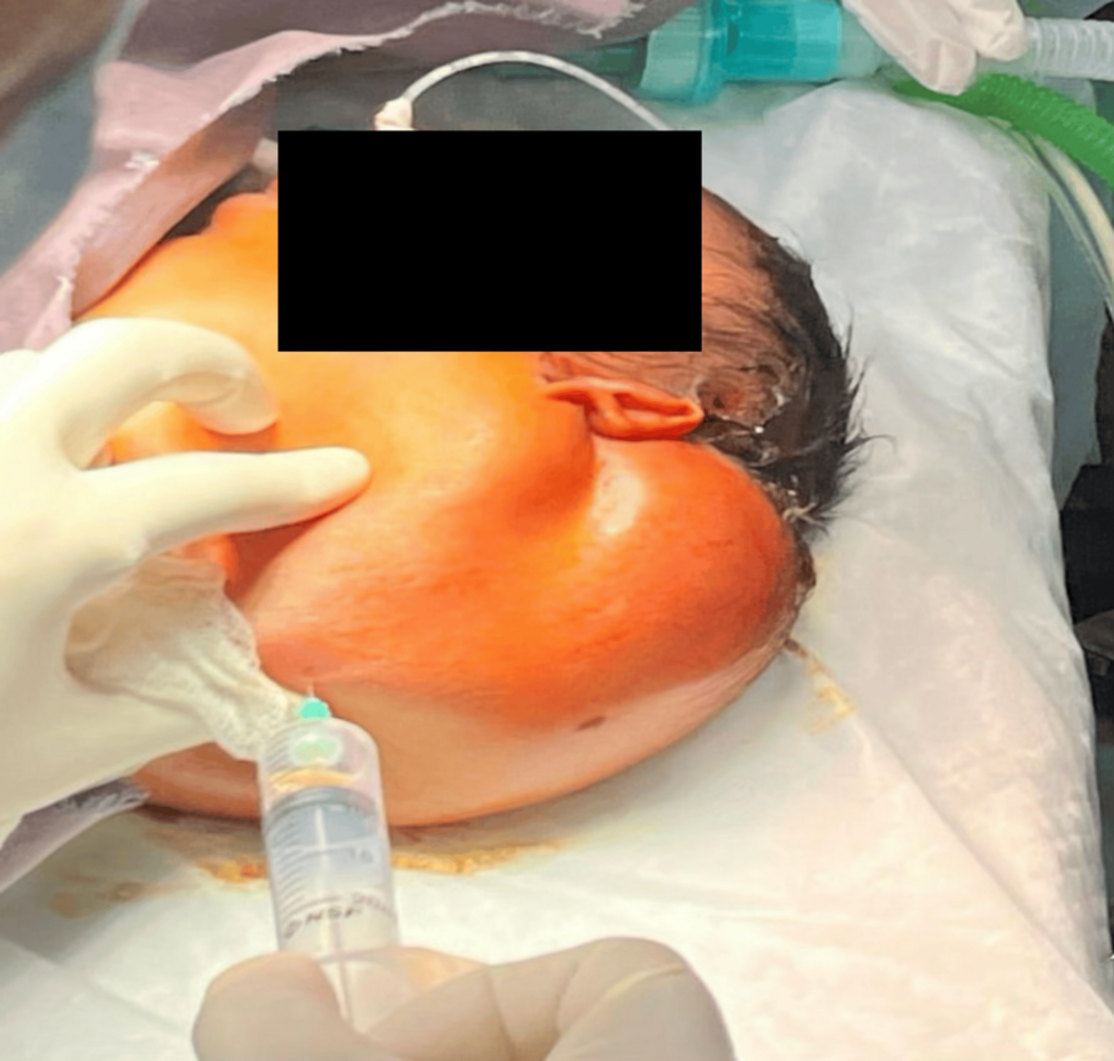
Intra-operative picture showing aspiration of contents followed by instillation of dose-adjusted diluted bleomycin.

Over the next 12 hours, the swelling progressively enlarged, resulting in tachypnea, stridor, and increased respiratory effort, indicative of impending airway obstruction. Considering the high surgical risk and imminent airway compromise, a decision was made to perform ultrasound-guided intralesional bleomycin sclerotherapy as an emergency salvage procedure. Following high-risk informed written consent, awake endotracheal intubation was performed to mitigate the risk of airway loss with complete paralysis. The cystic contents were aspirated, and bleomycin (0.5 mg/kg, within the recommended safe therapeutic dose, diluted in 20 mL of normal saline) was carefully injected into multiple loculations under aseptic conditions using ultrasound guidance. The patient was closely monitored for potential drug-related toxicities, including pulmonary, dermatologic, and hematologic effects, throughout the procedure and postoperatively. The procedure was well tolerated, with no immediate complications (Figure 2).

**Figure 3:**
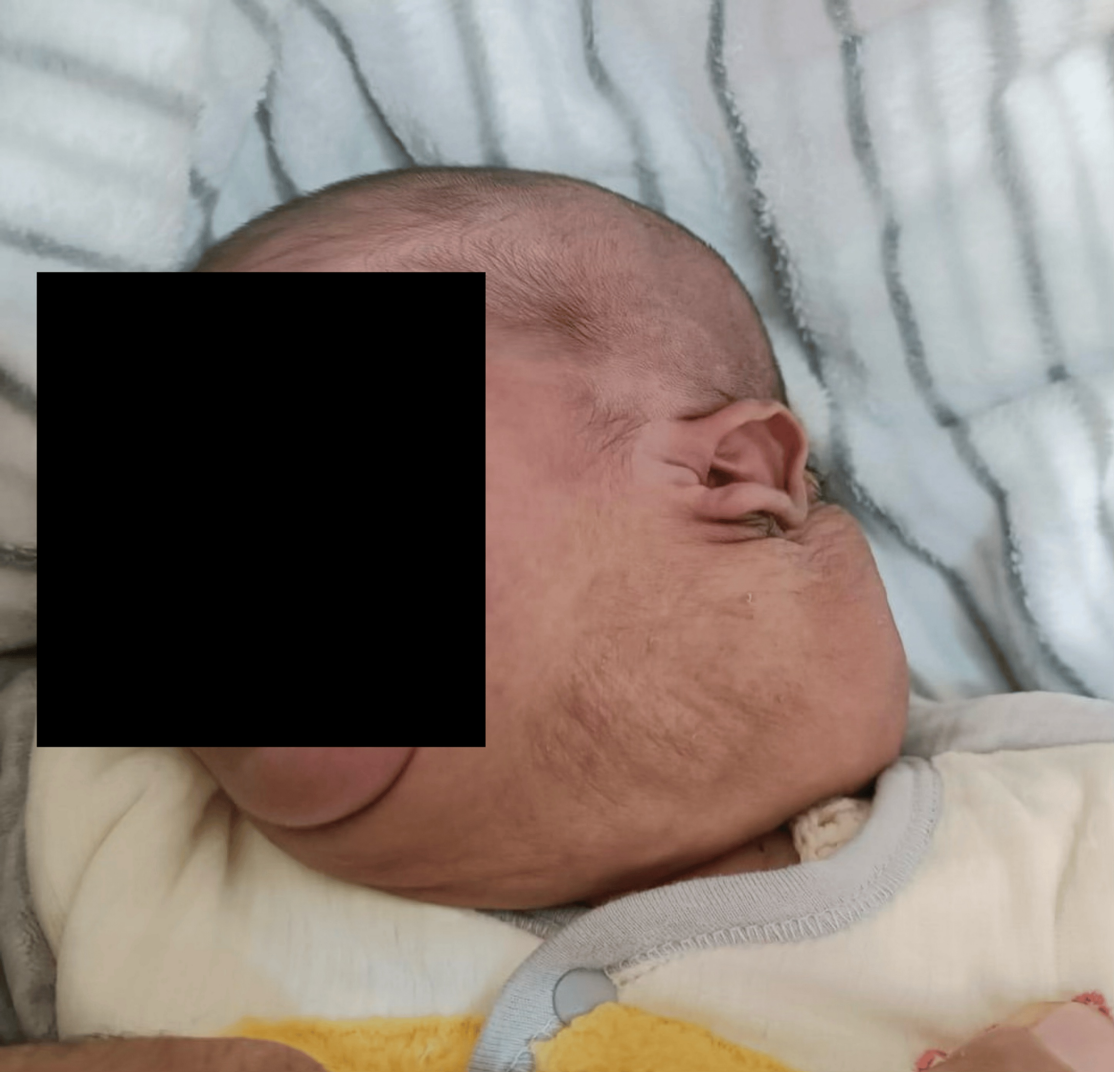
Significant reduction in swelling size on discharge after the first session of bleomycin sclerotherapy.

The child responded well to the emergency bleomycin sclerotherapy, with a marked reduction in the size of the swelling. He was discharged on full oral feeds on the fifth day of admission, with the residual swelling measuring 12 × 16 cm (Figure 3).

**Figure 4:**
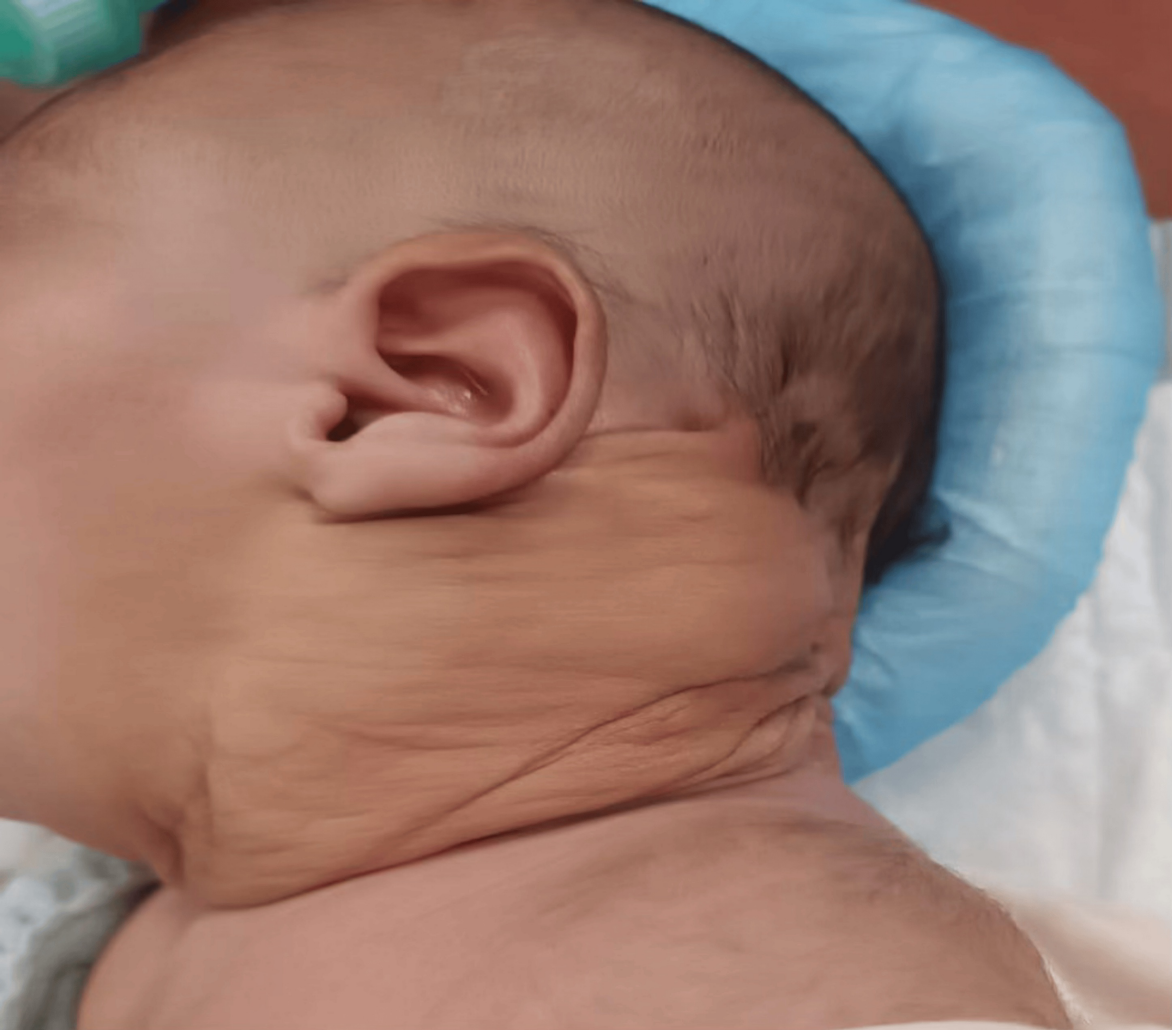
Swelling on 12-week follow-up showing good response to initial bleomycin sclerotherapy.

The child was followed up at two weekly interval which showed a more progressive and gradual decrease in swelling size. The child was kept under observation up to 12 weeks following the initial sclerotherapy session, when his swelling size was 8 x 10 cm with predominantly macrocytic component (Figure 4).

**Figure 5:**
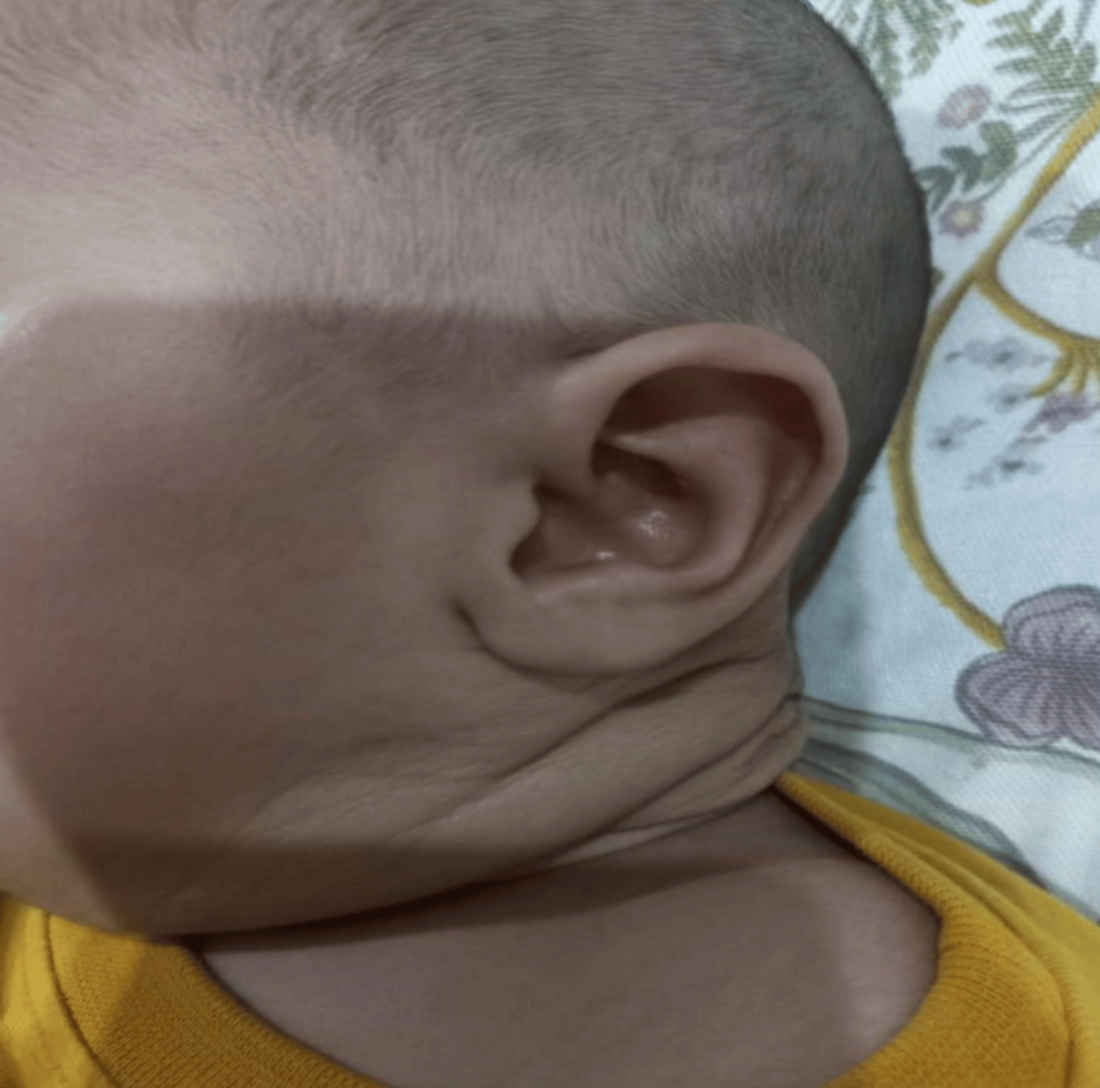
Follow-up showing complete resolution of neck swelling with redundant skin.

In view of the favorable response to the first sclerotherapy session, a second session of bleomycin was planned after counseling the parents regarding the procedure. The second bleomycin session was administered under ultrasound guidance at the same dose as the first session. The child had an uneventful recovery and was discharged on the third postoperative day. Serial follow-up at regular intervals demonstrated a further reduction in the size of the swelling, with complete resolution by eight weeks after the second sclerotherapy session, as confirmed by both clinical examination and neck ultrasonography. Only minimal residual skin laxity was noted on the left side of the neck; a plastic surgery opinion was considered but deemed unnecessary due to the minimal nature of the laxity (Figure 5).

## Discussion

Giant cervical cystic hygromas in neonates pose an immediate threat to the airway due to external compression and distortion of the laryngotracheal axis. Early airway stabilization followed by definitive lesion-directed therapy is therefore essential, as airway compromise remains the leading cause of early morbidity and mortality in these patients [6].

Although surgical excision has traditionally been considered the definitive treatment, neonatal surgery for extensive or infiltrative lesions is technically challenging and associated with significant risks, including massive hemorrhage, nerve injury, incomplete resection, and intraoperative airway loss. In scenarios where immediate surgery carries unacceptable risk, minimally invasive sclerotherapy offers an effective alternative by rapidly reducing cyst volume and alleviating mass effect while avoiding the hazards of emergency surgical intervention [7].

Several sclerosants have been used for intralesional therapy, including bleomycin, OK-432 (Picibanil), doxycycline, and ethanol. Among these, bleomycin is widely regarded as the preferred first-line agent in children due to its availability, cost-effectiveness, and consistently favorable outcomes in lesion regression [8].

Huang et al. reported a neonate with a giant macrocystic cervical hygroma in whom airway management was particularly challenging, emphasizing the importance of early intervention to prevent progressive respiratory compromise that may become fatal with lesion growth [9]. Similarly, Zeng et al. described a preterm, low-birth-weight neonate with a large prenatally diagnosed cervical cystic hygroma successfully managed with two sessions of injection sclerotherapy, resulting in complete lesion regression [10].

Our index case was unbooked and remained stable until the second day of life, when respiratory distress developed. Management with two sessions of intralesional bleomycin sclerotherapy administered eight weeks apart resulted in complete resolution. This highlights the critical importance of antenatal surveillance and planned delivery at a tertiary care center, as acute perinatal airway compromise can be rapidly fatal. Aljifri et al. further demonstrated the efficacy of bleomycin in a unique case managed with a combination of the ex utero intrapartum treatment (EXIT) procedure and repeated sclerotherapy sessions, achieving excellent functional and cosmetic outcomes [11].

While systemic bleomycin toxicity, particularly pulmonary fibrosis, remains a concern, pediatric experience suggests that low-dose intralesional administration across multiple sessions has a favorable safety profile [12]. Careful calculation of the bleomycin dose, aspiration of cystic contents prior to injection (similar to the PAIR technique (Puncture, Aspiration, Injection, Reaspiration)), and ultrasound guidance are crucial steps that significantly reduce the risk of intravascular spread and local complications [13]. Long-term follow-up studies indicate a durable reduction in macrocystic disease burden, as observed in our case; however, mixed and predominantly microcystic lesions are less responsive and may require multimodal therapy, including surgery with or without adjunctive sclerotherapy [14].

## Conclusions

Giant congenital cervical cystic hygromas in the neonatal period pose an immediate and potentially life-threatening risk to the airway, often necessitating urgent intervention. When surgical excision is considered high risk or technically challenging because of lesion extent or impending airway compromise, ultrasound-guided intralesional sclerotherapy offers a safe and effective alternative. In our case, early recognition of progressive airway compromise and timely bleomycin sclerotherapy successfully stabilized the neonate and resulted in significant lesion regression without complications. This experience highlights the importance of maintaining a high index of suspicion for imminent deterioration and supports sclerotherapy as a life-saving, minimally invasive strategy for managing critically located lymphatic malformations in neonates.
